# One Bane of Prudery

**Published:** 1911-11

**Authors:** G. Henri Bogart

**Affiliations:** Terre Haute, Indiana


THE
### TEXAS MEDICAL JOURNAL.
Established July, 1885

F. E. DANIEL, M. D., - - - - Editor, Publisher and Proprietor

Published Monthly.—Subscription, $1.00 a Year.

VOL. XXVII. AUSTIN, NOVEMBER, 1911. No. 5.

The publisher is not responsible for the views of the contributors.

For Texas Medical Journal.

### One Bane of Prudery.

DR. G. HENRI BOGART, TERRE HAUTE, INDIANA.

She was a proud social and intellectual leader in the little city, late past the fifty mark, unashamed to acknowledge her years, and when she asked me to step in and chat a bit, it was no unusual occurrence, and I was prepared to listen to a discussion of the last read book—usually some classic—or consideration of some phase of her club work.

But, when she asked me, an insistent tremor of pathos ringing through her voice, "Why is it that so many men, after a life of probity and virtue, when they pass life's divide, start the downward path morally as well?"

I paused for a moment to get my bearings.

It happened that her husband, who had been the average respectable man, had lately taken up with some of the commonest of the lewd women of the town, and that he was so unsophisticated, "green," that the small boys had caught on (boys nowadays know far more on sex matters than they are credited for, and that, too, mostly distorted, evil knowledge).

We, his familiar friends, were wondering how soon he would limp downtown, carrying a cane to ease an unexplained "sprain," the women at social functions would whisper among themselves when the wife entered, the while he stood as high as ever in the community, through the unconscious operation of the "double sex standard" lie.

I asked whether she wanted me to answer her question in platitudinary way, or did she want the full truth in seeming brutality of frankness.

"Oh," and the tears stood out through her tones, "tell me all, all."

I replied that false ideals are at the bottom of all this special form of misery, which is far more common than is usually believed.

The boy learns early, before he knows aught else of the function, that manhood is measured by sex prowess and virility; he hears foul tales, having for their sole feature this nasty lie, and with the passing of life's noon he is stricken with dread at the seeming waning of his sex power, his "manhood."

So ingrown is this falsity, that the gravest objection to the sterilization of the unfit is the statement, "You have no right to interfere with a degenerate's manhood (?)."

I use the word "seem" advisedly, for in reality the man whose sexual powers have been conserved along the lines of decency and physiological limits, while he may lose in frequency, will gain in the satisfaction of gratification sufficiently to make the compensating balance.

When, however, under the false ideal of life and the misleading teachings of prudery, he feels that he is failing, he will seek the stimulus of fresh fields of conquest. The word conquest is but another manifestation of the conventional falsity of prudery.

As the man's life is entering this danger zone, the wife usually alters her status of womanly power and inclination; her menopause has decreased the already weakened tenor of her nature; a weakness because that under the false social standard she has never had the function properly cultivated, and now, when he most needs the tender and careful ministration of the wife, in the fulfillment of the nuptial contract, he meets with indifference and apathy.

She interrupted me, "You are like all the men, you are harking back to Adam and trying to throw the blame onto the shoulders of the woman."

She is a notable housewife, her table linen is immaculate and dainty, her silver and china are both artistic and glittering with care, her menus are a never failing source of surprise and pleasure to those fortunate enough to be her guests.

I said to her, "You are notable for the elegance of your hospitality. Suppose that your husband were to come home, weary and hungry, and you were to go into the pantry and snatch handful after handful from roast, fruit, pastry, ices, to throw all into a dingy pan, then after pouring coffee and sauces over the mess, you were to toss the pan carelessly onto a bench with the words, 'There's your supper,' or that you should say, 'You know where to find the pantry, and if you want anything, help yourself.' How long, under such conditions, would the harmony of your home be preserved?"

"Why, he would have me before an insanity commission in short order," was her astonished reply.

"You would not blame him were he to take his meals at the hotel or the restaurant?"

"No, but I fail to see the connection."

Then I told her of the two great appetites which in reality rule the actions of the world of material folks—how I like that good old-fashioned word "folks."

I told her that the food desire is for the preservation of mankind in the selfish individual, while the sex hunger is for the preservation of the race. Both are proper, both are necessary, both are God-given.

The evolution of civilization has placed the former on a high esthetic plane, and relegated the other to a degradation where it is considered unmentionable.

Yet, the latter, the sex impulse, is the unselfish, the impersonal action. To prepare for it, to encourage its fruition, we have established the home, with all its aroma of clinging gladnesses, with its pure, sweet enticings of environment; we deck and beautify the body, with its residential mind, to make the person "good to look upon"; we have built up the grand, complex life habits of the world, and it is best to glorify the home whose cornerstone is marriage, and marriage is, per se, a contract, a God-given sacrament, intended, primarily, for the procreation of descendants, with whatever else of whim or wisdom the contracting parties may add foı its embellishment.

We sing, "What Is Home Without a Mother," and we debase even the thought of the propriety of discussing the act of motherhood. And the sexual relationship, for which all this is designed, which is its sole foundation (though not with our conscious volition), must not be considered, not talked about, nor even allowed to enter our thoughts, for "it is impure."

The canon law recognizes the fact, for it provides fully that the partnership may be dissolved for adultery, though it allows to the wife the power to condone the offense, but when one of the partners displays impotence, sterility, or the infection of the black plague, it takes from her this privilege of condonation, as the failure of the prime object of the marriage is an assault on the comity of the body politic.

Few of those who administer these laws, or who make statutes for its enforcement, recognize the basic principles upon which it is founded.

Woman has been taught that she should repress all womanly desires and intuitions until the long line of environments, which in the end become heredities, has actually fitted her into the groove of living and false standard, spontaneously.

She interrupted, "What has all this to do with my question?"

I told her that the wife, who most probably through all of her married life has failed both to herself and to her husband in nuptial duty, when his danger period appears becomes yet more lax. When he is most in need of her tender care, to protect him from himself then she carelessly sets her nuptial table with the scantiest of cold scraps, or else, with expressions of loathing and contempt, allows him to "help himself."

"What should she do?"

I made answer that the courtesan presents all the blandishments with which art and nature may equip her, as a matter of business, to attract her victims, precisely as the landlord serves food and pleasant surroundings for his hotel, and these the wife, in the citadel of home, should meet as part of homekeeping.

"You lose sight of the moral side of the question."

"The moral forces are presumed to be working all the time, and, really, I consider what I have just said to be a part of the moral issue, the keeping of a contract. The wife should render herself as attractive as does the lewd woman."

Indignant? Dear me, yes; her words fairly hissed, "Then you would have a good woman to resort to the wiles of the dissolute?"

"In a measure, yes; not only that, but more so. I would have her to eliminate all of the grossness, all of the counterfeit and at the same time to add all that true womanliness may be; for the sexual act, relieved from gross bestial horror, controlled and pure, actuated by love, is the sweetest, dearest, holiest relationship known to humanity, and should be approached with reverential attitude by both the conjugal pair, whom 'God hath joined together,' with full determination that it be complete and perfect."

"Then you are blaming me?"

"Please to explain."

"You have recognized that my question is personal, that I mean my own husband. Frankly and fully tell me how I may hold him, wherein I have failed in my duty."

I carefully gave her the desired information, and was much gratified when he, so far as the world of gossip could determine, returned to the allegiance of home and no more sought for "fresh ducks."

I wrote a short sketch of the case for a journal, and was astounded at the voluminous correspondence which ensued. Women whose physicians had suffered them to read the paper wrote asking "How," one woman wrote that it had saved her home from wreckage, and that she had copied the story to present her daughters at marriage, and the dean of a woman's college asked permission to incorporate it into a booklet for presentation to the graduates of the institution, which permission I gave her.

In view of these facts I am now giving the full report, with the explanation of the method to the profession.

My prescription was certainly ethical, it removed an atrophy of function, and gave to certain organs their full physiological action.

The Greeks, with their deep and delicate insight into life's philosophy, have one beautiful parable-myth, the marriage of Cupid (love) with Psyche (mind), the persecution of the married pair by Venus (mere amorousness) and the triumph of the marital joy when the mighty Jupiter welcomed them to Olympus and held the chalice of immortality to the lips of Psyche.

The sex appetite may be degraded precisely in the same manner as the food desire.

Try to picture some cave man of our common ancestry striking down his victim, tearing out the palpitating heart from its body and rending it with fiendish pleasure, while warm blood streamed from his ferocious features, adown his hairy breast. Contrast this with the courtly gentleman, with all the refinements of the amenities which we dub civilization as he partakes of an elaborate banquet, the while music and flowers and soulful companionship add the accessories of racial inspiration. We have developed to the utmost the amenities of the table, and it is well, but in the main we have neglected the more important function, relegating its consideration to ignominous disrepute.

The average man is prone to gratify his sexual appetite in manner similar to the savage of the illustration.

False ideals have held the woman to consider the manifestation of any desire, or pleasure of gratification as indecorous, as a necessary evil that she would fain dispense with were it not a forced concomitant of home-making. This training through the generations has become so quasi-normal that many live the lie; indeed, I know one sweet, pure woman who had borne two children before she had experienced orgasm, who came to me in alarm over the occurrence, and whom I was able to place in the path of true womanliness by a little advice.

Normally, sexual desire and power should be equal in the sexes. With many men, sad to say, we hark back to the era when woman was man's plaything and slave, a mere machine to gratify his lust, to be caged in a harem.

Conventionality today, with its hideous specter of the double standard of sexual morality, winks at the wrongdoing of the male; in fact, rather expects him to abandon the paths of virtue to some extent; while damning his sister, should even the shadow of reproach fall upon her—these bars are as cruel as the material iron of the harem window, while the foul tongue of gossip is a watchdog more implacable than the vile eunuch.

Few, very few, enter the marriage relation with but the faintest conception of the proper manner of procedure and accomplishment, and most are totally ignorant of its best purposes; their ignorance leads them through life with no more of the humanizing element than has the bloodsmeared savage, revelling in his gruesome tidbit.

Like some of my correspondents, you are asking "How?"

The first preparation should be mental and spiritual.

The pair should jointly approach the nuptial offering as a sacred rite.

Each should come, filled with a desire to minister to the joy of the other as the olden priest purified himself with the bath and garbed himself in spotless raiment, typifying spiritual purity. Physically, there should be leisurely interchange of endearments, and natural warmth is kindled into a steady glow, caressing and fondling until the blaze of the altar and not the lurid rage of the conflagration, the brilliancy of the arc and not the baleful glare of the lightning's flash; and with it all decent, calm decorum should prevail.

"Drink slowly, sip life's varied cup,
And taste it as you go;
The daintiest half of all they sup,
The hasty never know."

In the act itself the same aplomb should continue with gentleness and consideration.

There should be no undue haste, and as it usually happens that one of the pair is more active than the other, that one should exercise such restraint as shall insure that the supreme moment shall be mutual.

There should be no haste in breaking away from the embraces of love, the endearments should continue until the high tension of the physical system relaxes and the waves of transference of magnetic, or electrical, emanation shall have regained perfect balance.

Then will follow a glow of satiety, of love harmony that is indescribably happy, beautiful and soothing.

On the contrary, when the act is done violently, precipitately, there remains either a sensation of disgust and apathy or of craving for more indulgence, unfulfilled passion seeking cure—simila similibus curanter.

Such coition is equally harmful with masturbation, sometimes more so, when carried to its logical extremes with resulting excess, and is a prime cause of "lost manhood."

The man on whom this phase of the evil most operates is simply burned out with the rage of passion as the smith who overheats the forge destroys the iron when he goes beyond the welding heat.

It may be objected that the plan suggested is artificial; it is, and all of civilization is the same.

Because some olden ancestry of mine dwelt in a cleft of a cliff, or a hollow tree, robed himself insufficiently with skins, barks and grasses, and gorged to repletion when seed, roots or game came his way, and starved between times, is no reason why we should abandon our artificial houses, foods and garments with the other comforts of this era.

The sense of physical well being, of appeased desire, following indulgence as described, is insurance against the evil of excess.

The man whose taste has been brought to the perfection of proper embraces of the lovelit nuptial altar is immune to the allurements of the common drab who has no more fascination for him than has the female of the barnyard.

We declare reciprocal love to be as necessary to the perfection of the sexual act as is the other person.

Without this it is but a form of masturbation, and in proportion as the passions are the more excited, may be more baneful in result.

Lacking in such mutuality, the marriage altar becomes all too frequently but the theater of a series of rape assaults, legalized to be sure, but heinous beyond words for expression.

The perfection of the act as partially described fills the actors in the beautiful drama with the perfection of conjugal peace.

The converse conditions are best illustrated by the following facts, culled from the 400-page report of the Chicago Vice Commission.

A resort in that city was raided, and the books captured and made part of the court records. Each of the eighteen inmates was given credit on the day book for checks turned in, the rule of the place being that half of the dollar charged belonged to the inmate and the other half to the owner. Weekly summaries were made, and a monthly balance sheet was struck in the ledger. This record extended from August, 1908, to June, 1910, a period of twenty-two months, and showed that these eighteen girls had entertained 179,599 men, or an average of slightly more than fifteen each day for each inmate.

Another set of accounts showed that during a great influx of visitors to the city, a place convenient to the crowd had entertained sixty men each daily during the meeting. These figures and details may be found on pages 99-101 of the report mentioned.

Two pages earlier in the book is the record of a case that went to the Supreme Court, where a 16-year-old girl was popular with the hardened denizens of the low dive where but 50 cents was charged, for the books showed that her average day's work was the entertaining of twenty-six men and boys.

Such women, amid such a riot of debauchery, can neither know, nor yield any of the true pleasure of sexuality.

The barest thought of consorting with such a one would be horribly repulsive to the man accustomed to love's perfect union.

I was about to use the terms bestial and brutal, but pause to apologize to the brute, though she were the filthiest sow that ever revelled in a wallow.

With the most of the animals, coition is solely for the purpose of procreation, but with the human it is different.

As typified in the Greek myth, we have indication of the recognition of the fact that mind rules in the human animal.

The brute, not having the light of reason to control him, is protected by instinct, but with the man and woman the soul feeds upon this food of love, unless the mind allow the mere animal to overcome it, and the same act may be either the most elevating or the most debasing of all our material actions as we decide.

The knowledge and practice of sane, sweet conjugal connection would cement many a broken home, ease many an aching heart.

Every community has its homes wherein the under current of life is a diabolical travesty upon Edenic happiness, because of careless, ignorant, savage atavism of sexuality.

Doctor, your instruction may save many such.

The question of the social evil, with its resultant invasions of the black plague, is becoming of such moment as to prove a national menace and the best of beneficent intelligence is enlisting in earnest effort to combat it.

And with all this, the evil grows by leaps and bounds.

The social evil is "business"; it has its system of accounts and like other business it depends upon the inexorable law of supply and demand, and the demand of today is such that the natural sources of supply are insufficient and have to be forced with white slavery.

The man who associates is guilty, far more guilty than his degraded consort, since with him the act is voluntary, with her mainly compulsion, whether that force be an insufficient wage, drug-knockout drops, the strong arm or a traitorous courtship.

The moral and the legal forces have been marshalled against sexual crime since the Almighty slew Onan, and the horrible stream of filth pours in ever increasing volume into the placid purity of life's sea. Because these forces have not driven the horror out of existence is no reason why they should retreat from the firing line. When a great general knows his troops can not drive out a foe, he bids them hold the last ditch, while swift couriers hasten the reserves to their aid.

The best reserve in this combat of good and evil is open education that shall throw off the shackles of prudery and false modesty and teach purity as an element of personal well being and even as a matter of mercenary benefit as along the lines of communal economics, and private greed.

Each person who gives of his intensity to a given cause is prone to see that cause loom before him as the one great panacea. Thus the church, to whom belief is sufficient guide, expects thus to control the evil, the courts with their wealth of stringent statutes can not comprehend why the majesty of law has not swept the blot from the land, and the educator seeks to tell the erring of their wrong, and anticipates a millenium forthwith.

All three are honestly and earnestly trying to do all, and they are that much in error.

Some one said:

> "There is so much of bad in the best of us,
> There is so much of good in the worst of us,
> That it hardly becomes any one of us
> To talk about any one of the rest of us."

We need, all of us, to open our eyes a bit wider and to wipe out the moats from our moral vision so that we can see to recognize the other fellow as an ally, and not as a rival, and all work in harmony.

The man who has come to know and practice love's true acumen will no more seek the embraces of the painted woman than would the cultivated epicure rob the Digger Indian of his nasty dinner of squirming worms and noxious insects.

Did all married couples know and practice the sane, sweet and simple plan than I have outlined the mass total of human happiness would be advanced and the damning demand of the Moloch of society would be curtailed. I used the word Moloch advisedly; he was the god whose sacrifices consisted of the children in innocence, even as does the social evil.

Nor am I one of the reformers who declares his own hobby a panacea; it is only one of the weapons that may be used for purity.

The pioneer felled the forest, tree by tree, and each tree, chip by chip. Laws can only be enforced when the preponderance of public opinion demands enforcement, and every man won to the cause of right is one more unit in the balance, which must hang one way or the other. Prudery and false ideals of the sexual relation are only to be supplanted by honest endeavor, which at this time is shared by pure, earnest women, and the best intelligence of the medical profession.

American womanhood, having learned something of the fearful price that their innocent sisters are paying for the contamination of the monster, are banding and crying to the medical man, in tones choking with mingled blood and tears, "How? How?"

Shall we guide them with intelligent knowledge?

As a national characteristic we howl ourselves hoarse with songs which, whatever their merits or lack as poems, breathe deeply the beauties of motherhood, and it is well.

At the same time we frown darkly, frigidly on those who would think that we had allowed ourselves to think on maternity and conception or aught of the baby until it shall have been born, with all that prenatal influence and heredity may mean in their then unalterable coloration of the future left at haphazard.

We tell that "all the world loves a lover," we invest courtship and marriage with all the glamour of idealism and abuse him who dare hint at the natural cause and purpose of these things; we joy with Longfellow over the "hanging of the crane," and rejoice with the Puritan pair when we gaze on the picture of the children who came to crown the home, and we weep when life is barren of the sweet lullabys of babyland; yet we held up our hands in holy horror when Mrs. Wilcox sang:

"Come cuddle your head on my shoulder, dear,
Your head, like the golden rod,
And we'll drift together, away from here,
To the beautiful land of Nod."

Is it not time that the national genius shall give to the crowning glory of womanhood its own?

Shall we not rescue the consideration of sexuality from the filthy banal pool of scurrility and invest it with that sacred regard of which it is worthy?

Shall we not make of this, the most vitally holy relationship, a safeguard to the purity of the home life and one powerful factor in the elimination of the curse which will, if not controlled, sweep the best of the nation into the cesspool?

Let us as a race be honest, let us seek to educate the great mass mind of the nation away from the fraudulent filthiness of prudery and false modesty.

Let us teach the candidates for this priesthood of the nuptial altar that which shall enable them to fulfill the sacrament with a soul fully awake to the sacredness of woman's duty and man's obligations.

---

For Texas Medical Journal.

